# Diversity of malignancies in patients with different types of inborn errors of immunity

**DOI:** 10.1186/s13223-022-00747-2

**Published:** 2022-12-12

**Authors:** Marzieh Tavakol, Samaneh Delavari, Fereshte Salami, Sarina Ansari, Seyed Erfan Rasouli, Zahra Chavoshzadeh, Roya Sherkat, Hamid Ahanchian, Soheila Aleyasin, Hossein Esmaeilzadeh, Nasrin Moazzen, Alireza Shafiei, Farhad Abolnezhadian, Sara Iranparast, Sareh sadat Ebrahimi, Tannaz Moeini Shad, Salar Pashangzadeh, Farzad Nazari, Arezou Rezaei, Ali Saeedi-Boroujeni, Mohammad Nabavi, Saba Arshi, Morteza Fallahpour, Mohammad hassan Bemanian, Samin Sharafian, Sima Shokri, Sarvin Eshaghi, Shiva Nazari, Bibi Shahin Shamsian, Mehrdad Dargahi Mal-Amir, Roya Khazaei, Pooya Ashkevari, Armin Khavandegar, Sabahat Haghi, Marzie Esmaeili, Hassan Abolhassani, Nima Rezaei

**Affiliations:** 1 Non-Communicable Diseases Research Center, Alborz University of Medical Sciences, Karaj, Iran; 2 Research Center for Immunodeficiencies, Pediatrics Center of Excellence, Children’s Medical Center, Tehran University of Medical Sciences, Tehran, Iran; 3 Primary Immunodeficiency Diseases Network (PIDNet), Universal Scientific Education and Research Network (USERN), Tehran, Iran; 4 Pediatric Infections Research Center, Mofid Children’s Hospital, Shahid Beheshti University of Medical Sciences, Tehran, Iran; 5 Acquired Immunodeficiency Research Center, Isfahan University of Medical Sciences, Isfahan, Iran; 6 Clinical Research Development Unit of Akbar Hospital, Mashhad University of Medical Sciences, Mashhad, Iran; 7 Allergy Research Center, Shiraz University of Medical Sciences, Shiraz, Iran; 8 Department of Pediatric Immunology and Allergy, Namazi Hospital, Shiraz University of Medical Sciences, Shiraz, Iran; 9 Allergy Research Center, Mashhad University of Medical Sciences, Mashhad, Iran; 10 Department of Immunology, Bahrami Hospital, Tehran University of Medical Sciences, Tehran, Iran; 11 Department of Pediatrics, Abuzar Children’s Hospital, Ahvaz Jundishapur University of Medical Sciences, Ahvaz, Iran; 12 Department of Immunology, Faculty of Medicine, Ahvaz Jundishapur University of Medical Sciences, Ahvaz, Iran; 13 Student Research Committee, Ahvaz Jundishapur University of Medical Sciences, Ahvaz, Iran; 14 Department of Immunology and Allergy, Kerman University of Medical Sciences, Kerman, Iran; 15 Immunology Today, Universal Scientific Education and Research Network (USERN), Tehran, Iran; 16 Department of Microbiology, School of Medicine, Abadan University of Medical Sciences, Abadan, Iran; 17 Department of Allergy and Clinical Immunology, Rasool E Akram Hospital, Iran University of Medical Sciences, Tehran, Iran; 18 Student Research Committee, School of Medicine, Shahid Beheshti University of Medical Sciences, Tehran, Iran; 19 Pediatric Congenital Hematologic Disorders Research Center, Shahid Beheshti University of Medical Sciences, Tehran, Iran; 20 Pediatric Hematologist-Oncologist, Congenital Hematological Disorders Research Center, Mofid Children’s Hospital, Shahid Beheshti University of Medical Sciences, Tehran, Iran; 21 Department of Pulmonology, Faculty of Medicine, Ahvaz Jundishapur University of Medical Sciences, Ahvaz, Iran; 22 Department of Hematology and Oncology, School of Medicine, Alborz University of Medical Sciences, Karaj, Iran; 23 Division of Clinical Immunology, Department of Biosciences and Nutrition, Karolinska Institute, Stockholm, Sweden

**Keywords:** Primary immunodeficiency, Inborn errors of immunity, Malignancy, Hematologic cancers, Lymphoma, Leukemia, Solid tumors

## Abstract

Genetic defects in the development, maturation, and/or function of the immune cells can lead to Inborn errors of immunity (IEI) which may predispose patients to malignancies. The overall risk for cancer in children with IEI ranges from 4 to 25% and the type of malignancy is highly dependent on the specific mutant gene underlying IEI. We investigated 3056 IEI patients registered in the Iranian national registry between the years 1999 and 2020 in this retrospective cohort study. The frequency of malignancy and its association with the type of IEI in these patients were evaluated. A total of 82 IEI patients with malignancy were enrolled in this study. Among them, predominantly lymphoma was the most common type of malignancy (67.1%), followed by leukemia (11%), and cancers of the head and neck (7.3%). Among identified lymphoma cancers, non-Hodgkin’s lymphomas were the most frequent type (43.9%) followed by different subtypes of Hodgkin’s lymphoma (23.2%). Solid tumors (18.3%) appeared to be very heterogeneous by type and localization. The correlation between the type of malignancy and survival status and the association between the type of malignancy and IEI entities were unremarkable. The awareness of the association between the presence of IEI and cancer highlights the importance of a synergistic effort by oncologists and immunologists in the early diagnosis of malignancy and personalized therapeutic strategies in IEI patients.

**Supplementary Information:**

The online version contains supplementary material available at 10.1186/s13223-022-00747-2.

## Introduction

Primary immunodeficiency disorders (PIDs) or inborn errors of immunity (IEI) are a heterogeneous group of inherited disorders, usually associated with specific gene mutations [1, 2]. The overall incidence of IEIs has been reported to be 1 per 10,000 individuals [3]. Thanks to the advances in gene sequencing technologies, more than 450 gene defects associated with immunodeficiency disorders have been identified [4]. These disorders result in defects in the development and/or the function of one, and often more components of the immune system [5]. Moreover, these defects lead to reduced life expectancy in patients with IEI, in addition to diverse clinical manifestations including increased susceptibility to recurrent or prolonged infections, immune dysregulation phenotypes (such as severe atopy, allergy, autoimmunity, uncontrolled inflammation, lymphoproliferation), as well as predisposition to malignancies [6]. Malignancies are the second-leading cause of death in children and adults with IEI after infections [2, 7]. Given IEIs rarity, ascertaining the exact incidence rates of malignancies correlated with IEI is difficult. Nonetheless, the lifetime risk of cancer developing in children with IEI has been estimated to range from 4 to 25% [8, 9]. The most important reported mechanism of malignancy is compromised cell-mediated immunosurveillance and impaired immune function, which plays a significant role in protecting against tumors [10]. Other mechanisms are mostly associated with hematological malignancies including defects in DNA repair and impaired genetic stability, genetic predisposition, oncogenic viruses, persistent tissue inflammation and iatrogenic factors e.g., radiation [11].

The type of malignancy is highly dependent on the IEI subtype and the patient’s age [2, 12]. According to the previous reports, the most common IEIs associated with cancer are ataxia-telangiectasia (A-T), common variable immunodeficiency (CVID), as well as combined immunodeficiency (CID); furthermore, other PIDs were reported to be associated with malignancies, including severe combined immunodeficiency (SCID), agammaglobulinemia X-linked (XLA), and Wiskott-Aldrich syndrome (WAS) [12, 13]. In IEI cases, the overall incidence of hematological neoplasms is higher in rate than solid tumors. According to the database of the Immunodeficiency Cancer Registry (ICR), non-Hodgkin’s lymphoma (NHL) and Hodgkin’s lymphoma account for 48.6% and 10% of the malignancies in patients with IEI, respectively [12, 14]. Other types of cancer with excess relative risk in patients with IEI are gastric adenocarcinoma, thymoma, and rarely, melanoma, breast cancer, neuroblastoma, and medulloblastoma [14–16]. In the current study, we aimed to present a series of reported Iranian IEI patients with different types of malignancies.

### Patients and methods

For documentation, an evaluation sheet was developed to contain all patients’ demographic and laboratory data as well as clinical manifestations. We screened 3056 patients diagnosed with IEI in the Iranian PID registry (IPIDR) between the years 1999 and 2020 [17, 18]. The data of patients were obtained retrospectively from the IPIDR records. We assessed the demographic, immunological and clinical features of IEI patients with malignancies. Patients with incomplete data or those who did not meet the criteria were excluded. The diagnosis of IEI was done based on the European Society for Immunodeficiency (ESID) diagnostic criteria. The patients were categorized into IEI subcategories using the International Union of Immunological Societies (IUIS) classification [4].

We designed a comprehensive questionnaire that included; demographic information including the patients’ age at the first clinical presentation, the diagnosis and also the last follow-up visit, diagnostic delay; clinical complications such as infections, immune dysregulation, lymphoproliferative disorders and malignancies; laboratory tests data including the initial immunological work-up at the time of referral to our research center including the serum immunoglobulin titers, complete blood counts with differential, lymphocyte subsets and specific diagnostic tests for malignancies.

For each patient, cancer complications before and/or after diagnosis were recorded. The diagnosis of malignancy was based on international criteria by using clinical, radiological, laboratory and histological evidence. The evaluation for cancer diagnosis was reviewed for all patients by an oncologist and a subspecialist related to the affected organ. For each record, the date of cancer diagnosis and the topography and morphology codes were obtained (ICDO3, International Classification of Diseases, 10th revision [ICD-10]). Site-specific general population cancer rates were also obtained by 5-year age groups, sex, state, and calendar year.

Data were analyzed using the SPSS statistical software package version 25.0 (IBM corporation, Chicago, IL, USA). The Shapiro–Wilk test was used to validate the assumption of normality for a variable, and the nonparametric or parametric tests were carried out according to the normality assumption. A *p*-value < 0.05 was considered statistically significant. The association between nominal variables was analyzed using the Lambda correlation coefficient.

## Results

**Fig. 1:**
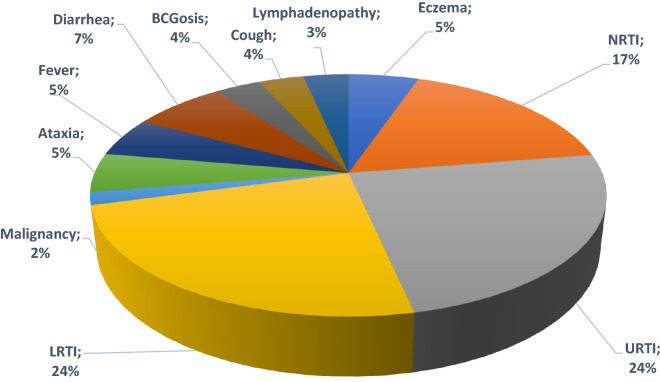
First presentation of IEI patients with malignancy.* URTI* upper respiratory tract infection,* LRTI* lower respiratory tract infection,* NRTI* non-respiratory tract infection

We analyzed data on malignancy types, clinical manifestations, and immunological findings from eighty-two IEI patients with malignancy (male to female ratio, 1.4:1, Tables 1, 2). The median age of the patients at the time of the study was 21 years. The youngest patient registered was 2 years old while the oldest one presented malignancy at 69 years of age. The median (interquartile range, IQR) of the onset of immunodeficiency symptoms was 3.25 (1.5–6.0) years. Also, the median (IQR) of diagnosis delay -the time between the onset of clinical features and diagnosis was 5.0 (4.0–7.0) years. From 82 patients, 27 cases (32.9%) were confirmed to be dead (15 males and 12 females), and 35 patients (42.7%) could not be located during the last 6 months of the study period. Among all 82 patients, the most frequent first clinical presentations were upper respiratory tract infections (17.1%) and lower respiratory tract infections (17.1%) followed by non-respiratory tract infections (12.2%). Other common first manifestations are indicated in Fig. 1. Of note, only one individual (1.2%) had been referred with malignancy as his first manifestation before the diagnosis of IEI.

**Table 1:** Clinical and immunologic data of IEI patients with different types of malignancies

Parameters	HL (n = 19)	NHL (n = 36)	Leukemia (n = 9)	Solid tumor (n = 15)	*p* value*
Clinical presentations					
Sex ratio (M/F)	9/10	20/16	6/3	10/5	0.28
Consanguinity (%)	8(42.1)	23(63.9)	3 (33.3)	11 (73.3)	0.78
Death (%)	8(42.1)	13(36.1)	2(22.2)	3(20.0)	0.20
Age at the onset of IEI, year, median (IQR)	5(0.5–7)	3(1–7)	3(0.75–48)	5.2(2.5–12.2)	0.21
Age at the diagnosis of malignancy, year, median (IQR)	17.3 (8.5–26.0)	14.0 (6.5–21.7)	15.2 (10.5–23)	18.5 (10.0–27.5)	0.38
Current age, year median (IQR)	25 (11–29)	17 (11–31)	18.5 (3.7–26.7)	25 (12–39)	0.21
Otitis media (%)	9 (47.4)	13 (36.1)	1 (11.1)	4 (26.7)	0.82
Sinusitis (%)	11 (57.9)	14 (38.9)	1 (11.1)	7 (46.7)	0.25
Pneumonia (%)	10 (52.6)	19 (52.8)	5 (55.6)	7 (46.7)	0.09
Bronchiectasis (%)	2 (10.5)	8 (22.2)	2 (22.2)	6 (40.0)	0.05*
Severe infections (%)	3 (15.8)	1 (2.7)	2 (22.2)	0	0.33
Chronic enteropathy (%)	3 (15.8)	4 (11.1)	0	6 (40.0)	0.003*
Failure to thrive (%)	3 (15.8)	8 (22.2)	1 (11.1)	4 (26.7)	0.17
Lymphoproliferation (%)	6 (31.6)	12 (33.3)	0	4 (26.7)	0.69
Autoimmunity (%)	6 (31.6)	5 (13.9)	2 (22.2)	2 (13.3)	0.62
Allergy and atopic diseases (%)	3 (15.8)	8 (22.2)	2 (22.2)	2 (13.3)	0.45

*p-value calculated to indicate significant differences between hematologic and solid tumors

**Table 2:** Immunologic lab data at the time of IEI diagnosis

Parameters	HL (n = 19)	NHL (n = 36)	Leukemia (n = 9)	Solid tumor (n = 15)	*p* value*
Leukocyte/µl, median (IQR)	7600 (6565–9900)	6900 (3800–9630)	4400 (3100–13,340)	7100 (4290–12,600)	0.83
Lymphocyte/µl, median (IQR)	2721 (2412–4054)	2184 (1329–3651)	3080 (2137–26,904)	772 (510–2612)	0.05*
Neutrophils/µl, median (IQR)	3984 (2809–5527)	3608 (2933–7121)	1800 (691–73,402)	3900 (2940–5428)	0.85
Absolute T cells/µl, median (IQR)	2217 (1448–2856)	1519 (763–1936)	2022 (1167–2791)	464 (355–665)	0.09
Absolute T4 cells/µl, median (IQR)	552 (265–957)	325 (134–989)	606 (457–864)	238 (230–340)	0.14
Absolute T8 cells/µl, median (IQR)	2217 (1448–2856)	1519 (763–1936)	2022 (1167–2791)	464 (355–665)	0.08
Absolute NK cells/µl, median (IQR)	118 (102–129)	231 (80–693)	313 (125–452)	170 (68–219)	0.26
Absolute B cells/µl, median (IQR)	134 (46–295)	183 (68–359)	225 (123–540)	38 (20–110)	0.07
Serum IgG mg/dl, median (IQR)	587 (339–993)	300 (71–640)	130 (13.7–517)	400 (46–1360)	0.93
Serum IgA mg/dl, median (IQR)	28 (3.5–84)	8 (2–40)	16.5 (3.5–44)	16 (4–37.5)	0.92
Serum IgM mg/dl, median (IQR)	49 (15–123)	41 (20–117)	24 (8.5–64)	67 (22–122)	0.65
Serum IgE IU/ml, median (IQR)	3 (0.07–14)	25 (0.9–226)	1 (0.5–1.5)	10 (4.8–11,705)	0.35

*p-value calculated to indicate significant differences between hematologic and solid tumors

No malignancy was reported in patients with complement deficiency, phagocyte disorders and autoinflammatory diseases. Therefore, patients were classified into five main groups of IEI according to the IUIS classification. The majority of cases in the present cohort had predominantly antibody deficiencies (PAD), clinically diagnosed in 50 patients (61%), followed by syndromic combined immunodeficiency (CID) in 20 patients (24.4%), defects in intrinsic and innate immunity in 5 patients (6.1%), diseases of immune dysregulations in 3 patients (3.7%), non-syndromic CID in 3 patients (3.7%) and, there was one patient with unclassified IEI (1.2%) (Additional file 1: Fig. S1).

Among all PAD patients enrolled in this study, the most prevalent disorder was CVID in 38 cases (71%), followed by agammaglobulinemia in 7 cases (14%), selective antibody deficiency (SAD) in 2 patients (4%), hyper-IgM syndrome (HIGM) in 2 patients (4%) and, IgAD in only one patient (2%). Among CVID patients, the most prevalent malignancy was lymphoma in 27 cases (71.1%), followed by leukemia in 3 cases (7.9%), solid tumor in 5 patients (13.2%) and, benign epithelial neoplasm in only one patient (2.6). For patients diagnosed with agammaglobulinemia and malignancy, lymphoma (57.1%) and leukemia (42.9%) were the principal malignancies reported.

The cancer patients with syndromic CID consisted of A-T in 15 patients (75%), hyper-IgE syndrome (HIES) in 4 patients (20%) and, Nijmegen breakage syndrome in only one case (5%). The most prevalent malignancy in A-T patients was lymphoma (66.7%), and then solid tumor in 3 cases (20%). Additionally, in HIES patients, lymphoma was presented in 3 cases (75%).

Patients with innate immunodeficiency (5 cases) included chronic mucocutaneous candidiasis (CMCC) in three patients (60%) and, Mendelian susceptibility to mycobacterial diseases (MSMD) in two (40%). The type of malignancy in CMCC patients was solid tumor (2 out of 3 patients), and in two MSMD patients, solid tumor and lymphoma were reported. Two autoimmune lymphoproliferative syndromes (ALPS) patients (2 out of 3 patients) with lymphoma, and one patient (1 out of 3 patients) with regulatory T cell defect were reported in the immune dysregulation cluster. Immunodeficiencies affecting cellular and humoral immunity involved only three cases (3.7%) of leaky and hypomorphic combined immunodeficiency (CID). As the above data suggests, not all these immunodeficiencies presented an equal susceptibility to the development of cancer.

**Fig. 2:**
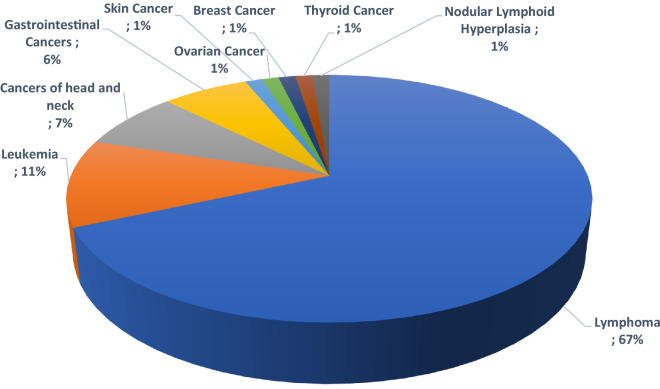
Type of Malignancy in a cohort of 82 IEI patients

**Fig. 3:**
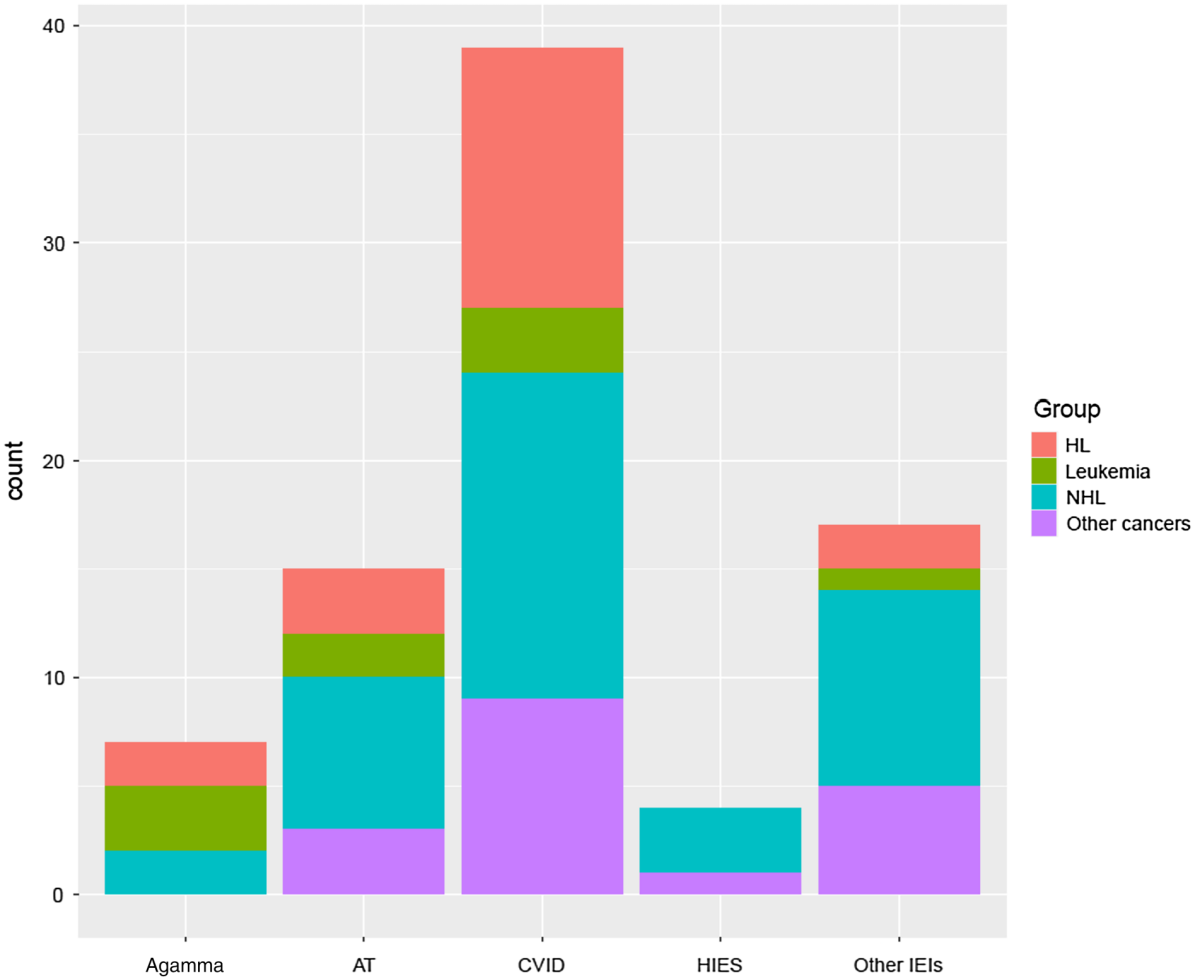
Frequency of type of malignancy based on the types of IEI

Among our 82 IEI patients with malignancy, predominantly lymphoma was the most common type of malignancy, constituting 67.1% of patients (n = 55, Figs. 2, 3, Additional file 1: Fig. S1), followed by leukemia 11% (n = 9), cancers of the head and neck 7.3% (n = 6), gastrointestinal cancers 6.1% (n = 5), skin cancer 1.2% (n = 1), ovarian cancer 1.2% (n = 1), breast cancer 1.2% (n = 1), thyroid cancer 1.2% (n = 1) and, nodular lymphoid hyperplasia 1.2% (n = 1). In addition, for 3 patients the pathological evaluation was not conclusive regarding the type of cancer. Among hematological cancers (78%), non-Hodgkin’s lymphoma was the most frequent type with 36 patients (43.9%) followed by different subtypes of Hodgkin lymphoma with 19 patients (23.2%) and leukemia with 9 patients (11%). Solid tumors (18.3%) appeared to be very heterogeneous by type and localization as follows: 5 cases of head and neck tumors (2 squamous cell carcinoma of tongue, 1 mandibular squamous cell carcinoma, and 2 brain tumor), 5 cases of gastric tumors (4 gastric adenocarcinomas, 1 colorectal cancer), one case of skin cancer (1 squamous cell carcinoma), one case of ovarian cancer (1 ovarian cystadenoma), one case of breast cancer, one case of thyroid cancer (1 thymoma), and one case of nodular lymphoid hyperplasia (Figs. 2, 3) Interestingly the pattern of immunologic data were distinct in IEI patients with solid tumors mainly due to leukopenia, reduced T-and B cell subsets at the time of IEI diagnosis (Table 2). Moreover, the assessment of solid tumors in comparison with hematological cancers demonstrated a significantly higher incidence of bronchiectasis and chronic enteropathy in solid tumors (*p* = 0.05 and 0.003, respectively) (Table 1).

**Fig. 4:**
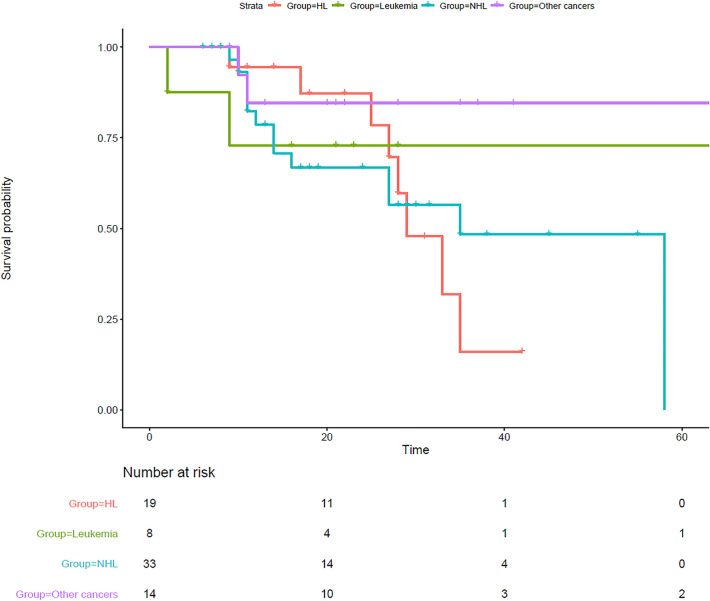
Survival analysis of cohort of 82 IEI patients with malignancies based on the type of cancers

**Fig. 5:**
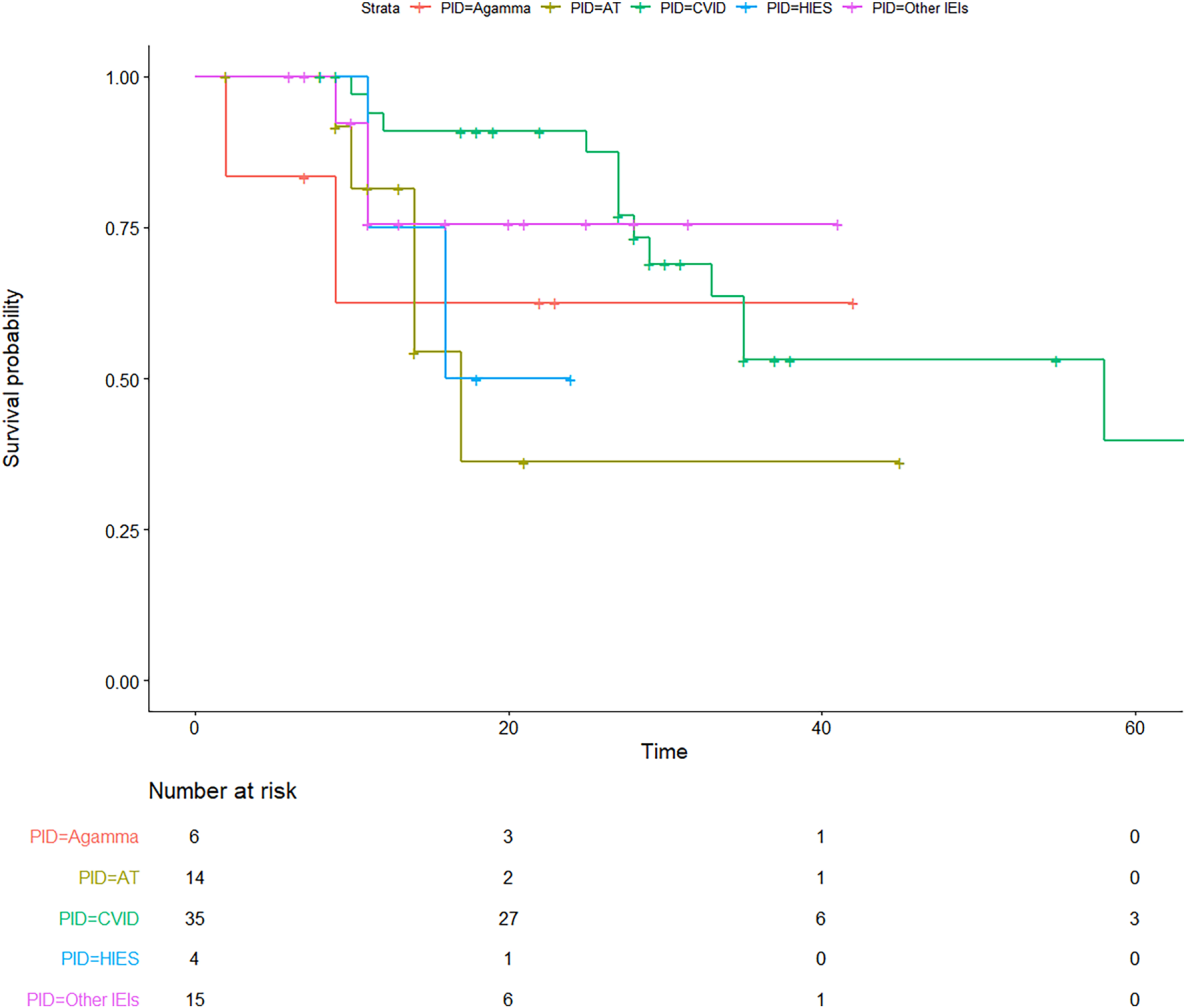
Survival analysis of cohort of 82 IEI patients with malignancies based on the type of immunodeficiency

To evaluate the association between the type of malignancy and survival status, the Lambda correlation coefficient was used. The Kaplan Meier survival analysis of IEI patients with malignancy showed an increased mortality rate in the patients with lymphoma (mainly Hodgkin’s lymphoma, Fig. 4) and also a worse survival rate in IEI patients with AT compared to other patients with malignancies (Fig. 5; Additional file 1: Fig. S2).

## Discussion

In this study, we evaluated the overall incidence of different types of malignancy in 82 subjects with IEI enrolled in the IPIDR. The age of patients at the time of the study ranged from 2 to 69 years, with a median age of 19.6 years, which was similar to those derived from the ICR (Immunodeficiency Cancer Registry), whose average age was 20 years [19]. In this cohort, there was a male predominance (58.5%), in the line with other studies in the literature [13]. The result can be justified by the fact that many types of IEI (XLA, WAS, XSCID) have an X-linked transmission which is only observed among males. Moreover, the differences in the male to female ratio in other studies may be attributed to the heterogeneity of the immunodeficiencies distribution in various populations. In addition, the occurrence of malignancy in subtypes of IEI can be regarded as the consequence of different distributions of the IEI subtypes in the population, the variability of the carcinogen pathogens exposure, and genetic variations and type of mutation which affect the individual’s susceptibility to the development of the tumor resulting in differences in cancer development in various IEI subtypes [13, 20]. Within the four main categories of IEI associated with cancer in our study including CVID, agammaglobulinemia, AT and HIES several lines of evidence have been highlighted for the underlying mechanisms of tumorigenesis. Antibody deficient patients mainly with defective PI3K or NFKB pathways can develop several different cancer predisposing means including avoiding immune destruction, resisting cell death, inducing angiogenesis, deregulating cellular energies, activating invasion and metastasis, tumor promoting inflammation, and enabling replicative immortality. Moreover, cases with syndromic CID due to DNA repair can increase genome instability and mutation and evade growth suppression. HIES is also prone to sustaining proliferative signaling [10].

Several studies have reported the incidence of malignancies among IEI patients. Among them, Maffeis et al. (2019) reported the incidence of tumors among 690 patients with IEI, diagnosed from 1990 until 2017 in Brescia, in which, 25 out of 690 patients (3.6%) developed 33 tumors. Of the 25 affected patients, 8 patients suffered from common variable immunodeficiency (CVID), 5 from CID, 3 AT, 2 from Hermansky-Pudlak type 2 (HSP2), 2 XLA, 2 from WAS, 2 from HIES, and 1 from SCID [13]. Our data appear to confirm what emerges from the major studies in the literature, in which the incidence of hematological neoplasms was higher than solid tumors in IEI patients. Also, Mayor et al. evaluated the incidence of cancer in subjects with IEI, enrolled in the United States Immune Deficiency Network (USIDNET) registry. They reported 171 separate cancers in the cohort of 3,658 IEI patients (4.7%). Ninety-one cancers were diagnosed in women and 80 cancers in men. Of the total cancers diagnosed, 82 (48%) were lymphoma, 25 (15%) were skin cancers, 14 (8%) were genitourinary, 14 (8%) were gastrointestinal cancers, 10 (6%) were breast cancers, 9 (5%) were endocrine cancers, 6 (4%) were cancers of the head and neck, 5 (3%) were lung cancers, 2 (1%) were bone cancers, and 4 (2%) were cancers with unspecified origins [21]. Additionally, in Maffeis et al. (2019) study, hematological malignancies were prevalent (22/33, 66.7%) with a minority of solid tumors (11/33, 33.33%). In particular, non-Hodgkin’s lymphomas were the most frequent (16/33, 48.48%) [13].

Our data indicated that different types of malignancy could be associated with specific types of IEI. Therefore, education of physicians about the risk of malignancies in IEI, patient monitoring and considering underlying IEI as a probable cause of malignancy in children and adolescents will optimize the diagnostic process, personalized treatment, and ultimately improve management and, eventually survival of patients. The molecular diagnosis of these IEI patients with different types of cancers should be conducted using next generation sequencing in future studies as an important step toward targeted management. Moreover, a prospective study with a larger number of patients would fully elucidate the association between malignancy and type of IEI.

## Supplementary Information

**Additional file 1: Fig. S1**: Frequency of type of malignancy based on the IUIS classification. Table 1: Immunodeficiencies affecting cellular and humoral immunity, Table 2: Combined immunodeficiencies with associated or syndromic features, Table 3: Predominantly antibody deficiencies, Table 4: Diseases of immune dysregulation, Table 6: Defects in intrinsic and innate immunity. **Fig. S2**: Survival analysis of cohort of 82 IEI patients with malignancies based on the IUIS classification. Table 1: Immunodeficiencies affecting cellular and humoral immunity, Table 2: Combined immunodeficiencies with associated or syndromic features, Table 3: Predominantly antibody deficiencies, Table 4: Diseases of immune dysregulation, Table 6: Defects in intrinsic and innate immunity.

## Data Availability

Not applicable.
