# Supplementary material for: Diversity of malignancies in patients with different types of inborn errors of immunity
Source: Allergy Asthma Clin Immunol. 2022 Dec 12;18:106. doi: 10.1186/s13223-022-00747-2 (PMC9743521; doi:10.1186/s13223-022-00747-2)
Supplement: Supplementary file 1 — Additional file 1: Fig. S1: Frequency of type of malignancy based on the IUIS classification. Table 1: Immunodeficiencies affecting cellular and humoral immunity, Table 2: Combined immunodeficiencies with associated or syndromic features, Table 3: Predominantly antibody deficiencies, Table 4: Diseases of immune dysregulation, Table 6: Defects in intrinsic and innate immunity. Fig. S2: Survival analysis of cohort of 82 IEI patients with malignancies based on the IUIS classification. Table 1: Immunodeficiencies affecting cellular and humoral immunity, Table 2: Combined immunodeficiencies with associated or syndromic features, Table 3: Predominantly antibody deficiencies, Table 4: Diseases of immune dysregulation, Table 6: Defects in intrinsic and innate immunity. [file 13223_2022_747_MOESM1_ESM.docx]

**Additional file 1**

**Figure S1: Frequency of type of malignancy based on the IUIS classification.** Table 1: Immunodeficiencies affecting cellular and humoral immunity, Table 2: Combined immunodeficiencies with associated or syndromic features, Table 3: Predominantly antibody deficiencies, Table 4: Diseases of immune dysregulation, Table 6: Defects in intrinsic and innate immunity.

**Figure S2: Survival analysis of cohort of 82 IEI patients with malignancies based on the IUIS classification.** Table 1: Immunodeficiencies affecting cellular and humoral immunity, Table 2: Combined immunodeficiencies with associated or syndromic features, Table 3: Predominantly antibody deficiencies, Table 4: Diseases of immune dysregulation, Table 6: Defects in intrinsic and innate immunity.
